# Alteration in Redox Status and Lipoprotein Profile in COVID-19 Patients with Mild, Moderate, and Severe Pneumonia

**DOI:** 10.1155/2022/8067857

**Published:** 2022-11-14

**Authors:** Miodrag Lalosevic, Jelena Kotur-Stevuljevic, Jelena Vekic, Manfredi Rizzo, Tijana Kosanovic, Iva Perovic Blagojevic, Aleksandra Zeljkovic, Danilo Jeremic, Marija Mihajlovic, Aleksa Petkovic, Lejla Hajdarpasic, Marjana Djordjevic, Violeta Dobrilovic, Sanja Erceg, Sanja Vujcic, Jelena Marjanovic, Jovana Milijic Jovanovic, Jovica Saponjski, Natasa Bogavac-Stanojevic

**Affiliations:** ^1^Radiology Department, University Hospital “Dr. Dragisa Misovic-Dedinje”, Belgrade, Serbia; ^2^Department of Medical Biochemistry, University of Belgrade-Faculty of Pharmacy, Belgrade, Serbia; ^3^Department of Health Promotion, Mother and Child Care, Internal Medicine, and Medical Specialties, University of Palermo, Palermo, Italy; ^4^Department of Laboratory Diagnostic, University Hospital “Dr. Dragisa Misovic-Dedinje”, Belgrade, Serbia; ^5^Orthopedics Department, Institute for Orthopedic Surgery “Banjica”, Belgrade, Serbia; ^6^Cardiology Clinic, University Clinical Centre of Serbia, Belgrade, Serbia

## Abstract

**Background:**

Metabolic alterations, particularly disorders of lipoprotein metabolism in COVID-19, may affect the course and outcome of the disease. This study aims at evaluating the lipoprotein profile and redox status in SARS-CoV-2 infected patients with different pneumonia severity and their association with lethal outcomes.

**Methods:**

The prospective cohort study was performed on 98 COVID-19 patients with mild, moderate, and severe pneumonia. Lipid and inflammatory parameters, lipoprotein subclasses, and redox status biomarkers were determined at the study entry and after one week.

**Results:**

Compared to patients with mild and moderate pneumonia, severely ill patients had higher oxidised low-density lipoprotein (oxLDL) and malondialdehyde levels and lower high-density lipoprotein cholesterol (HDL-C) concentrations and paraoxonase 1 activity. Reduction in the proportion of large HDL 2a subclasses with a concomitant increase in the proportion of smallest HDL 3c and small dense LDL (sdLDL) particles was observed in patients with severe disease during the time. However, these changes were reversed in the mild and moderate groups. The results showed a positive association between changes in oxLDL and total antioxidative status. However, prooxidants and antioxidants in plasma were lower in patients with lethal outcomes.

**Conclusions:**

Increased levels of oxLDL and sdLDL particles may contribute to the severity of COVID-19. The role of oxidative stress should be clarified in further studies, mainly its association with lethal outcomes.

## 1. Introduction

COVID-19 pandemic caused by SARS-CoV-2 virus still represents a central public health concern [1]. Patients with SARS-CoV-2 infection can experience clinically diverse manifestations, from no symptoms to critical illness. It has been recognised that poor COVID-19 prognosis is associated with alterations in the host immunological status, increased proinflammatory cytokines levels, age and concomitant cardiovascular, and other comorbidities [2]. Bearing in mind the role of lipid profile in the pathogenesis of cardiovascular diseases, more recently, metabolic alterations, particularly disorders of lipoprotein metabolism in COVID-19, have been increasingly recognised [3]. It was reported that total cholesterol (TC), low-density lipoprotein cholesterol (LDL-C), and high-density lipoprotein cholesterol (HDL-C) concentrations in COVID-19 patients decreased significantly compared to healthy individuals [4]. Another study showed a relationship between reduced LDL-C and HDL-C levels and the severity of COVID-19 disease [5]. However, in a recent meta-analysis, it was found that dyslipidemia increases the risk of severe COVID-19 [6] and may affect the outcome of COVID-19 [7]. Considering our previous findings that the preponderance of small HDL particles is associated with increased cardiovascular mortality [8], it is reasonable to assume that dysfunctional small HDL could be additional factor that contribute to lethal COVID-19 outcomes. Furthermore, evidence emerges that one of the most important factors in COVID-19 progression could be oxidative stress mediators influencing the redox balance of a cell [9]. In this sense, Martin-Fernandez et al. [10] first confirmed this hypothesis, presenting findings that patients with SARS-CoV-2 infection experience decreased antioxidant activity and increased damage at the cellular level.

Moreover, some studies reported increased prooxidants and biomarkers of lipid peroxidation in the blood of SARS-CoV-2 infected patients [9]. This excessive production of reactive oxygen species and predominance of dysfunctional HDL and small dense LDL particles (sdLDL) may result in the generation of oxidised LDL (oxLDL) particles [11]. OxLDL particles are directly involved in the progression of inflammation by increased accumulation in macrophages and the ability to promote the generation of reactive oxygen species, proinflammatory mediators, and procoagulant factors [12]. However, there is a lack of reports on oxLDL levels and changes in HDL and LDL subclasses distribution and their contribution to the severity of COVID-19. In line with the previous, the aim of the present study was to analyse the alteration of redox status and the lipoprotein profile in SARS-CoV-2 infected patients with different pneumonia severity and their relationship with lethal outcomes.

## 2. Material and Methods

### 2.1. Patients

The prospective cohort study was performed at the Clinical Hospital Center, Dr Dragisa Misovic-Dedinje, from July 2020 to October 2021. It encompassed 98 COVID-19 patients with positive results on the real-time fluorescent RT-polymerase chain reaction performed according to WHO guidelines [13]. For patients were recorded data regarding comorbidities such as cardiovascular disease (CVD), diabetes mellitus (DM), thyroid dysfunction and cancer, and data about regular therapy and treatments for SARS-CoV-2 infection. The anesthesiologist monitored patients and introduced oxygenated support when needed. Patients with the highest oxygen flow but with declining oxygen saturation were intubated. Of the 98 patients, 82 were hospitalised; 62 needed oxygenated support, and 40 needed ventilator support. Unenhanced computerised tomography (CT) scans were done for all patients using the Canon (former Toshiba), Aquillion One (TSX-301C), and 320-row MDCT System (Canon, Tokyo, Japan). Two radiologists reviewed all CT images with extensive experience in thoracic imaging on the diagnostic workstation (Vitrea extend-Vital, Canon, Tokyo, Japan) with multiplanar reconstruction (MPR) tools. The consensus of acceptance by both radiologists resolved all disagreements between them. Disease severity was determined based on lung involvement and relevant CT scoring [14]. According to this score, patients were divided into three groups: mild (score < 8), moderate (score between 8 and 18), and severe (score > 18) pneumonia. The study was conducted in accordance with the Declaration of Helsinki and approved by the Ethics Committee of Clinical Hospital Center, Dr Dragisa Misovic-Dedinje (No. 01-7661, dated July 1, 2020).

### 2.2. Laboratory Analyses

Blood was sampled (the first time point) the day after CT scoring. Since the usual time for recovery from the progressive disease stage is seven days [14], blood was collected after a follow-up period of seven days (the second time point). Immediately upon venipuncture, the samples were centrifuged, and plasma and serum were separated and stored at -80°C until analyses. TC, triglycerides (TG), HDL-C, and C-reactive protein (CRP) concentrations were determined using commercially available enzymatic and turbidimetric tests on Dimension EXL 200 (Siemens Healthcare GmbH, Germany) analyser. The concentration of LDL-C was calculated by the Friedewald formula. Interleukine-6 (IL-6) was determined on Immulite 2000 (Siemens Healthcare GmbH, Germany) immunoanalyser using a chemiluminescent immunoassay (CLIA). The level of oxLDL was measured by ELISA (Elabscience Biotechnology Inc., Houston, United States). Total antioxidant status (TAS), total oxidant status (TOS), superoxide dismutase (SOD), paraoxonase 1 (PON1), prooxidant-antioxidant balance (PAB), and total protein sulfhydryl groups (SH-groups) were measured on ILab 300+ (Instrumentation Laboratory, Milan, Italy) and described and published elsewhere [15]. The levels of superoxide anion (O_2_^−^) and malondialdehyde (MDA) were measured using methods developed by Auclair and Voisin [16] and Girotti et al. [17], respectively. Plasma HDL and LDL particles were separated using a nondenaturing 3-31% polyacrylamide gradient gel electrophoresis method. The LDL and HDL particle size determination and subclasses analyses were determined based on migration distance of the major peaks in densitometric profile, from calibration curve made from high molecular weight protein standards, carboxylated polystyrene microsphere beads, and human plasma with two LDL subclasses of known diameters. Relative proportions of LDL and HDL subclasses were determined by analysing areas under the peaks that correspond to the known ranges of particle diameters: LDL I (27.2-28.5 nm), LDL II (25.5-27.2 nm), LDL III (24.2-25.5 nm), LDL IV (22.0-24.2 nm), HDL 2b (9.7-12.0 nm), HDL 2a (8.8-9.7 nm), HDL 3a (8.2-8.8 nm), HDL 3b (7.8-8.2 nm), and HDL 3c (7.2-7.8 nm) [18].

### 2.3. Statistical Analysis

Data distribution was tested using the Kolmogorov-Smirnov test. ANOVA with Tukey's post hoc test (for normally distributed data) and the Kruskal-Wallis with Mann–Whitney *U* test with Bonferroni correction for subgroup differences (for skewed data) were used for mild, moderate, and severe group data comparison. Student's *t*-test and Mann–Whitney *U* test were used to test data differences between deceased and recovered patients. Categorical variables were tested by the Chi-square test. A two-way repeated measured ANOVA test was utilised to evaluate the effect of the time (changes in two repeated observations—the first and the second point) and the effect of time-outcome interaction for normally distributed data. We used Wilcoxon signed-rank to test whether the changes of skewed data in two repeated observations in deceased and recovered patients were statistically significant. Data are shown as mean ± standard deviation for normally distributed variables. The median for independent data or the median of difference for dependent data with interquartile range were presented for nonnormally distributed variables. Relative or absolute frequencies are shown for categorical variables. Moreover, to analyse whether the changes of examined parameters between the two-time points are interrelated, univariate and multivariate linear fixed-effect panel model regression was used. Cox regression was employed to model time to in-hospital death. The data were presented as hazard ratios with a 95% confidence interval (CI). The statistical analyses were performed with PASW® Statistic v. 27 (Chicago, Illinois, USA) software. A two-tailed *p* value ≤0.05 was considered to indicate statistical significance.

## 3. Results

Table 1 presents the clinical, radiology, and laboratory findings of 98 patients classified into mild, moderate, and severe pneumonia groups. No difference in gender distribution between the groups was found, but the patients with severe pneumonia were older than those with mild and moderate disease severity. Comorbid conditions before SARS-CoV-2 infection, such as CVD and DM, were more prevalent among patients with severe pneumonia. Typical symptom in patients with severe pneumonia was coughing or chest pain. According to the national COVID-19 protocol, all patients with moderate and severe pneumonia were hospitalised, while the hospital admission rate in patients with mild pneumonia was 54%. As expected, the median length of stay (LOS) in the hospital gradually increased with the disease severity. The patients with severe pneumonia had significantly lower oxygen saturation (SpO_2_) and received high-flow oxygen therapy. One patient with moderate pneumonia and one-third of patients with severe pneumonia required intubation. Severely ill patients received anticoagulant therapy, including aspirin (100%), fraxiparine (82%), and corticosteroids (97%). Besides anticoagulants, some patients with mild and moderate pneumonia also received antiviral medication. All patients were supplemented by vitamins C and D.

Table 2 shows laboratory parameters according to the disease severity. Evaluation of lipid status parameters showed that HDL-C concentrations were significantly reduced in severely ill patients compared to those with mild pneumonia. In addition, the patients with severe disease conditions had the highest oxLDL concentrations. Analysis of lipoprotein subclasses profile showed that patients with moderate pneumonia had higher proportions of small LDL III and HDL3b particles than patients with mild pneumonia. Compared to patients with mild and moderate pneumonia, severely ill patients had higher CRP and MDA but lower plasma prooxidants TOS and O_2_^−^ and antioxidants TAS, SOD, and PON1 levels. Additional differences were found between severe and mild pneumonia patients; those with severe pneumonia had higher IL-6 and PAB levels and lower total SH-groups.

**Figure 1:**
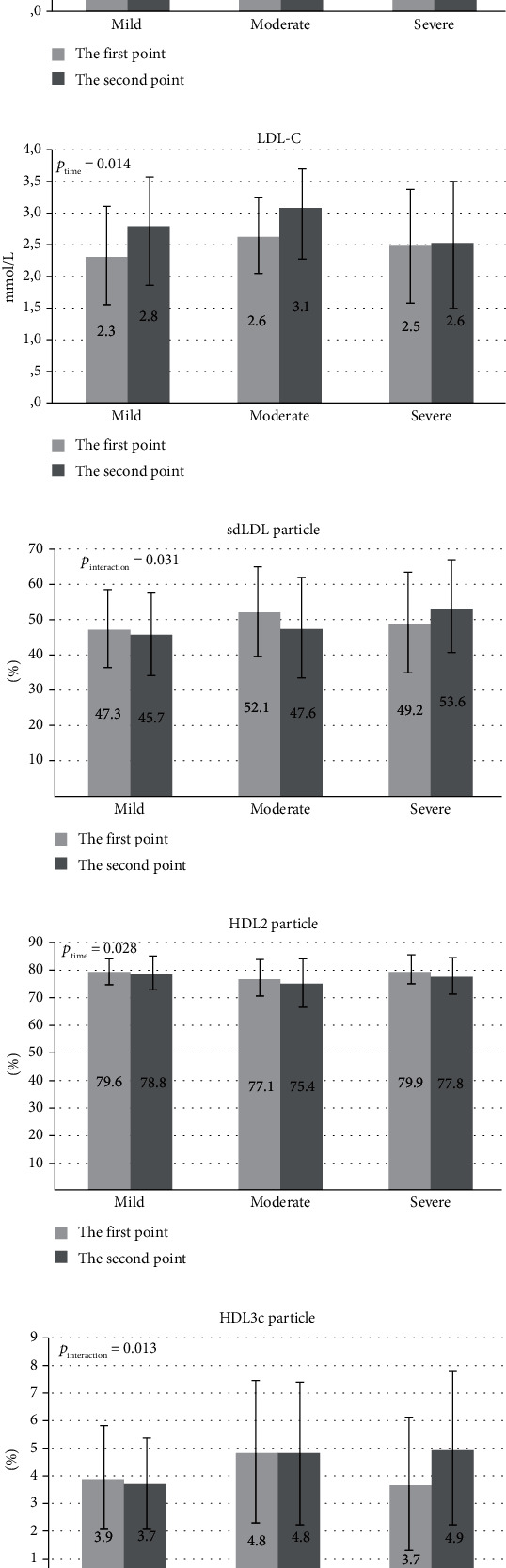
Changes of normally distributed lipid parameters after seven days of follow up. (a) Changes of TC concentration. (b) Changes of HDL-C concentration. (c) Changes of LDL-C concentration. (d) Changes of the proportion of sdLDL particles. (e) Changes of the proportion of HDL 2 particles. (f) Changes of the proportion of HDL 3c particles. (g) Changes of the proportion of HDL 2a particles.

We further analysed changes of examined parameters after one week. The results demonstrated that TC, HDL-C, and LDL-C concentrations increased in all three groups. At the same time, we observed a reduction of HDL 2 subclasses proportion in all groups. A significant interaction between disease severity and time was demonstrated for the relative proportion of HDL 3c and HDL 2a subclasses as well as the proportion of sdLDL particles. The proportions of HDL 3c subclasses and sdLDL particles decreased in mild and moderate pneumonia patients but increased in those with severe disease. In contrast, the proportion of HDL 2a particles increased in patients with mild and moderate illness and declined in patients with severe pneumonia (Figure 1).

**Figure 2:**
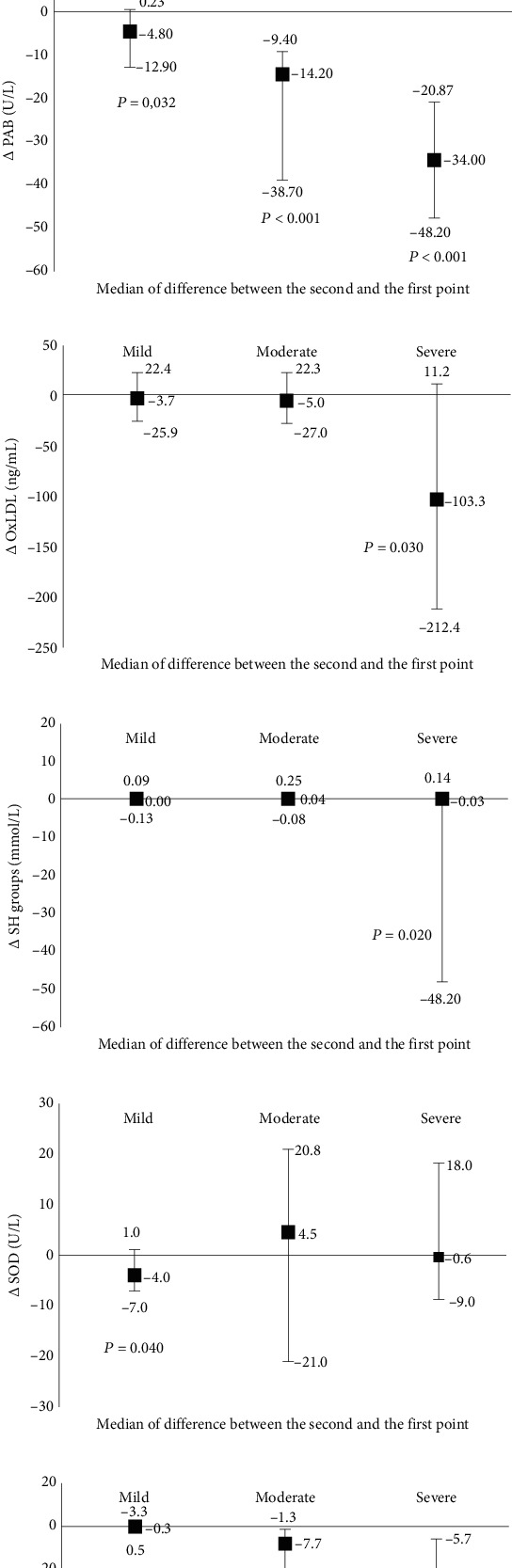
Changes of parameters with skewed distribution after seven days of follow up. (a) Changes of MDA concentrations. (b) Changes of TOS concentrations. (c) Changes of PAB activity. (d) Changes of oxLDL concentrations. (e) Changes of SH-groups concentrations. (f) Changes of SOD activity. (g) Changes of CRP concentrations. (h) Changes of IL-6 concentration.

The median of the difference between the two-time points revealed that PAB activity, IL-6, and CRP concentrations significantly decreased in severely and moderately ill patients. A significant reduction of total SH-groups and oxLDL concentrations was demonstrated in severely ill patients with a concomitant increase in TOS. In the patients with mild pneumonia, only SOD activity was decreased, and MDA was increased over time (Figure 2).

During the follow-up period, 18 patients (20%) died.  Table 3 presented significantly different laboratory and clinical parameters between deceased and recovered patients. Seventeen deceased patients had severe, and one patient had moderate disease conditions. Deceased patients were older and had significantly higher inflammatory parameters, oxLDL, and MDA. Also, LOS in hospital and oxygen supplementation rates was higher, but SpO_2_ was lower in deceased patients. Both prooxidants and antioxidants in plasma were lower in patients with lethal outcomes.

Cox regression analysis was employed to analyse the association between the examined laboratory parameter levels in the first point and the risk of fatal outcomes. The results showed that the association between higher oxLDL concentrations and increased mortality (hazard ratio per 1 ng/mL, 1.002; 95% CI, 1.000 to 1.005) was borderline significant (*p* = 0.05).

Moreover, we tested whether changes in laboratory parameters between the two-time points are associated with the oxLDL levels changes (Table 4). It was revealed that the rise in oxLDL concentration during follow up was accompanied by increase in PAB and TAS concentrations and a reduction in the proportion of HDL 3c subclasses. Next, we added time-varying PAB, TAS, and HDL 3c variables in the model. The model showed that an increase of 1 unit in TAS leads to a statistically significant increase in oxLDL concentration independently of other confounders.

## 4. Discussion

This prospective study revealed that alterations of HDL subclasses distribution, sdLDL particles, oxLDL, oxidants, and antioxidants levels contributed to the progression of COVID-19 disease. Additionally, both prooxidants and antioxidants were lower in patients with lethal outcomes.

In general, SARS-CoV-2 infection is associated with reduced HDL-C levels, while the concentrations of total and LDL-C are usually unchanged or slightly decreased [19]. Our current study confirms these findings, demonstrating the most significant reduction of HDL-C levels in patients with severe disease. In line with the previous, our severely ill patients had significantly reduced PON1 activity, indicating the compromised antioxidative potential of HDL particles. Existing literature suggests a marked decrease in HDL-C concentrations during the acute phase response due to suppressed apolipoprotein A-I (apoA-I) synthesis by proinflammatory cytokines. Also, the accumulation of acute-phase proteins, especially serum amyloid A, within HDL further reduces its apoA-I content [20]. Such profound modifications of HDL proteome are also associated with the loss of the antioxidative enzyme PON1, which ultimately leads to HDL dysfunction, as seen in cardiometabolic [21] and malignant diseases [22] but also in COVID-19 [23]. So far, data on HDL particles distribution in COVID-19 has remained scarce. A small pilot study by Ballout et al. [24] was the first that investigated NMR-based lipoprotein subclasses profile in severe COVID-19 patients. The authors found a significant reduction of HDL particles number, especially small HDLs, on hospital admission. Our current study further extends previous findings by demonstrating a substantial reduction in the proportion of large HDL 2a subclasses, with a concomitant increase of the smallest HDL 3c particles during the hospitalisation of patients with severe disease. It should be stressed that Ballout et al. [24] reported that a third of patients with severe COVID-19 had reduced concentrations of small-sized LDL particles. Similarly, in our current study, the patients with moderate rather than severe COVID-19 had the highest proportion of small LDL III particles. However, during the follow-up period, the proportion of sdLDL particles in patients with mild and moderate pneumonia significantly decreased, supporting a recent hypothesis by Schmelter et al. [25] that individuals' metabolic status might contribute to the course of COVID-19. Namely, they showed that the lipoprotein profile of asymptomatic anti-SARS-CoV-2 antibody-positive patients was similar to that of healthy controls. Interestingly, antibody titers against SARS-CoV-2 correlated inversely with small-sized LDL particles. Based on these data, Schmelter et al. suggested that better metabolic health could be associated with milder forms of COVID-19 [25]. Our current data support this observation, suggesting that metabolic health is equally important for reestablishing lipid homeostasis and possibly earlier recovery from COVID-19.

In contrast to the mild and moderate groups, our patients with severe COVID-19 experienced a significant increase in sdLDL particles over time. The preponderance of sdLDL particles is a firmly established risk factor for the development of atherosclerotic CVD and increased cardiovascular mortality [26]. Obesity and type 2 DM, two of the most frequent comorbidities associated with poor COVID-19 outcomes, are intimately linked with atherogenic dyslipidemia. Of note, every fifth patient in our severe disease group had diabetes, so it remains to be established whether sdLDL has a causal relationship with the adverse disease outcome and may serve as a therapeutic target or is a biomarker of severe COVID-19. Available data suggest that dyslipidemia worsens the course of COVID-19, while dyslipidemia treatment by statins seems to be associated with lower mortality [7]. However, since the patients included in the current study did not receive lipid-lowering medications, future studies are needed to address the possible favorable impact of statin therapy on COVID-19 prognosis. Nevertheless, disorders of lipoprotein subclasses distribution presented in our severely ill patients imply a predisposition for cardiovascular complications of COVID-19 survivors. Therefore, lipid screening and possible assessment of sdLDL particles could be advised for convalescents from severe disease, especially those with preexisting comorbidities.

Evidently, our patients with COVID-19 are in a state of severe immune system activation, which in turn causes oxidative stress burden. Olwal et al. [27] elaborated several similarities between sepsis and COVID-19 regarding pathophysiological and clinical characteristics. Keeping in mind the finding of Bojić et al. [28] of a significant fall in PON1 activities in sepsis patients, which was able to predict patients' mortality, we supposed that similar mechanisms could be responsible for the severe form of COVID-19 infection so as in sepsis. However, our severely ill patients and deceased ones had paradoxically significantly lower TOS and O_2_^−^ compared to mild and moderate group, so as to recovered patients, respectively. This decrease in prooxidant levels (O_2_^−^, H_2_O_2_, and lipid hydroperoxides, the last two measured through the TOS test) could be explained by a probable rise in reducing blood components (bilirubin, urea, and creatinine) which are capable of changing and neutralising prooxidants chemically. Mentioned blood reductants rise is well known in severely ill patients, sepsis and COVID-19 [29, 30]. However, it is unclear whether this increase is a consequence of impaired renal and/or liver function in critically ill patients or a generalised inflammatory state. In addition, exacerbated and uncontrolled oxygenation due to pneumonia can affect mitochondrial metabolism and the production of oxygen radicals [31]. Although oxidative stress is one of the causes of morbidity and mortality in COVID-19, our study did not find an association between redox status parameters and risk for the lethal outcome.

On the other hand, increase in oxLDL could be explained by PON1 protective role exhaustion documented here as a significant fall in this enzyme activity but also as a consequence of fast LDL lipid-hydroperoxides decomposition to their end products such as MDA and hydroxy-nonennal and other toxic aldehydes [32]. The proposed mechanism of rapid lipid-hydroperoxides degradation to their end-products explains the significant reduction in TOS concentration reported here in severe, so as in deceased patients' groups. Namely, lipid-hydroperoxides are markers of the early phase of oxidative stress, and they represent the main component of TOS (besides H_2_O_2_) [33]. Considering previously demonstrated mutual involvement of inflammation and oxidative stress in systemic hyperinflammtory state, i.e., cytokine storm, which is followed by the oxidative stress storm [34], it is reasonable to think about antioxidant supplementation. The patients in our current study had received vitamin C in dose of 2 g, according to the hospital treatment protocol. Therefore, we could speculate that noticed fall in superoxide anion and TOS levels was caused by vitamin C infusion. In line with previous notions, increased oxLDL concentrations in our COVID-19 patients are expected. Recent studies reported that oxLDL particles are directly involved in COVID-19 disease severity and lethal outcome by an increased accumulation in macrophages and the ability to promote the generation of reactive oxygen species and proinflammatory mediators [12]. In SARS-CoV-2 infected patients, oxLDL-stimulated macrophages promote the activation of the inflammasome and subsequent release of proinflammatory cytokines, such as IL-1B and IL18 that exacerbate tissue injury. On the other side, protective type I interferons production is suppressed [35]. Moreover, oxLDL, thought activation of oxidised LDL scavenge receptor (LOX-1), causes additional impairment of cell and tissue function [36]. Our study confirms these findings, showing the highest level of oxLDL in severely ill and deceased patients. In line with the previous, current results indicate that increased oxLDL concentration during the follow up might contribute to a lethal COVID-19 prognosis. However, studies with larger sample sizes are needed to clarify the association between oxLDL and lethal outcomes.

Additionally, changes in oxLDL were strongly influenced by alterations in antioxidant activity. The positive association between oxLDL and TAS changes throughout time suggests that prooxidants can stimulate total antioxidant capacity. On the other hand, an increase in antioxidant enzymes may be insufficient for cell and tissue protection [10]. Yet, we did not find any relation between changes in oxLDL and CRP and IL-6 levels, but we observed a high inflammatory profile in severely ill patients. Although IL-6 and CRP concentrations decreased after seven days of follow up, the levels of both biomarkers increased in patients with lethal outcomes. Consequently, we assume that a decrease in their levels may indicate future recovery of critically and moderately ill patients. Another important finding of this study was connected with the total SH groups content, which was significantly lower in the group of severely ill patients, compared to the mild pneumonia group. In addition, a significant fall in total SH groups level was found in severely ill patients during follow up. These unwanted changes in sulfhydryl reducing power of blood suggested an urgent need for antioxidants, SH groups donors, such as NAC or glutathione (GSH), because blood situation mirrors cellular GSH deficit and/or increased needs for GSH in severely ill COVID-19 patients. Intervention with NAC in COVID-19 patients was already published and justified elsewhere [37].

Some limitations of our study should be taken into account when comparing our data with the results of other studies—the most significant being differences in ethnicity, age, and prevalence of comorbid conditions among studied COVID-19 patients. Also, the sampling time and follow-up period should be considered when comparing prognostic information based on lipid and lipoprotein data across different studies. It should also be acknowledged that the vast majority of our patients with severe COVID-19 received corticosteroid therapy, which might be responsible for adverse modulation of LDL and HDL subclasses distribution observed in this group during follow up. However, corticosteroid treatment is commonly associated with increased TG levels [38]. Still, we did not find any difference in its levels between mild, moderate, and severe COVID-19 patients at baseline or significant changes over time among the groups. Because of SARS-CoV-2 complications, many of the patients admitted to the hospital could not give consent for participation in the study, and consequently, the sample was relatively small.

## 5. Conclusions

In conclusion, our study results indicated rapid changes in the lipid profile during the hospitalisation of COVID-19 patients, especially levels of oxLDL and sdLDL particles, which increase may contribute to the severity of the disease. Further studies should clarify the role of oxidative stress in the disease severity and prognosis.

## Figures and Tables

**Table 1:** Demographic and clinical characteristics of SARS-CoV-2 infected patients with different disease severity in the first point.

Parameter	Mild *n* (28)	Moderate *n* (23)	Severe *n* (39)	*p*
Male gender, *n* (%)	11 (39%)	12 (52%)	25 (64%)	0.132
Age, years	43 (35-51)	58 (47-64)^a£^	61 (47-68) ^a£^	0.001
Comorbidities				
CVD, *n* (%)	0 (0%)	1 (4%)	17 (44%)	<0.001
DM, *n* (%)	0 (0%)	0 (0%)	7 (18%)	0.007
Thyroid dysfunction, *n* (%)	0 (0%)	0 (0%)	4 (10%)	0.065
Cancer, *n* (%)	0 (0%)	0 (0%)	3 (10%)	0.131
COVID-19 symptoms and management				
Diarrhea, *n* (%)	1 (0%)	3 (10%)	5 (10%)	0.382
Cough or chest pain, *n* (%)	12 (43%)	14 (64%)	37 (95%)	<0.001
Hospitalisation, *n* (%)	15 (54%)	22 (96%)	39 (100%)	<0.001
LOS in hospital, days	4.5 (0-10)	10 (8-15)^a£^	18 (13-21)^ab£^	<0.001
Death, *n* (%)	0 (0%)	1 (4%)	17 (44%)	<0.001
Oxygen treatment				
SpO_2_, %	97 (95-98)	96 (95-97)^a£^	87 (78-91)^ab£^	<0.001
Oxygen supplementation, L/min	0	3 (0-5)^a£^	30 (15-40)^ab£^	<0.001
Intubation, *n* (%)	0 (0%)	1 (5%)	13 (36%)	<0.001
Therapy				
Fraxiparine, *n* (%)	13 (46%)	21 (91%)	32 (82%)	<0.001
Aspirin, *n* (%)	12 (43%)	4 (17%)	39 (100%)	<0.001
Favipiravir, *n* (%)	8 (29%)	4 (17%)	0 (0%)	0.003
Corticosteroids, *n* (%)	1 (4%)	4 (17%)	37 (97%)	<0.001

Categorical data are presented as absolute frequencies (percentages) and analysed by the Chi-square test. *P* ≤ 0.050 was considered statistically significant. Continuous data are presented as median and interquartile range and compared by the Kruskal-Wallis test. (a) Statistially significant compared to the mild disease group. (b) Statistically significant compared to moderate disease group. (£) *p* ≤ 0.017 was considered statistically significant (Mann–Whitney *U* test with Bonferroni correction).

**Table 2:** Lipid, inflammatory and oxidative and antioxidative stress parameters in SARS-CoV-2 infected patients with different disease severity in the first time point.

Parameter	Mild *n* (28)	Moderate *n* (23)	Severe *n* (39)	*p*
TC, mmol/L	4.20 ± 0.77	4.42 ± 0.81	4.07 ± 1.24	0.580
TG, mmol/L	1.53 (1.14-1.94)	1.64 (1.18-2.75)	1.70 (1.20-2.30)	0.585
HDL-C, mmol/L	1.14 ± 0.34	0.93 ± 0.28	0.90 ± 0.29^a¥^	0.016
LDL-C, mmol/L	2.33 ± 0.78	2.56 ± 0.63	2.45 ± 0.90	0.739
oxLDL, ng/mL	205 (177-234)	240 (198-275)^a£^	519.8 (297-688)^ab£^	<0.001
LDL I, %	22.1 ± 9.8	16.5 ± 7.5	20.6 ± 8.5	0.068
LDL II, %	30.6 ± 8.0	31.5 ± 8.1	30.3 ± 9.5	0.886
LDL III, %	21.2 ± 6.5	27.2 ± 8.7^a¥^	25.0 ± 9.6	0.024
LDL IV, %	26.2 ± 7.1	24.3 ± 6.8	24.1 ± 10.9	0.624
sdLDL, %	47.3 ± 11.1	52.1 ± 12.7	49.1 ± 14.0	0.423
HDL 2b, %	56.5 ± 6.8	54.0 ± 7.0	55.0 ± 5.5	0.365
HDL 2a, %	23.1 ± 3.2	23.0 ± 2.4	25.0 ± 4.6	0.057
HDL 3a, %	11.8 ± 2.9	12.0 ± 2.6	11.0 ± 2.2	0.310
HDL 3b, %	4.8 ± 1.6	6.2 ± 2.2^a¥^	4.8 ± 2.0	0.016
HDL 3c, %	3.9 ± 1.9	4.8 ± 2.6	3.6 ± 2.4	0.149
HDL 2, %	79.5 ± 4.6	77.1 ± 6.2	80.0 ± 5.5	0.109
CRP, mg/L	2.65 (0.80-8.68)	8.60 (3.50-50.60)^a£^	58.10 (27.30-135.70)^ab£^	<0.001
IL-6, pg/mL	3.60 (2.72-5.78)	5.40 (3.30-13.70)	12.40 (6.80-37.60)^a£^	<0.001
TAS, mmol/L	1030 (882-1140)	1084 (940-1137)	823 (563-1022)^ab£^	0.006
TOS, mmol/L	30.3 (22.8-40.8)	28.9 (23.5-41.0)	12.80 (9.8-19.6)^ab£^	<0.001
SOD, U/L	146 (141-152)	144 (135-181)	103 (94-125)^ab£^	<0.001
PON1, U/L	342 (218-812)	277 (184-643)	181 (115-545)^a£^	0.036
O_2_^.-^, *μ*mol NBT/min/L	25.0 (19.0-32.0)	24.0 (18.5-36.0)	14.0 (1.3-21.0)^ab£^	0.001
PAB, U/L	82.4 (63.0-94.3)	97.2 (86.5-113.5)^a£^	104.8 (91.4-125.4)^a£^	<0.001
MDA, *μ*mol/L	0.411 (0.356-0.523)	0.497 (0.390-0.629)	1.260 (1.193-1.433)^ab£^	<0.001
SH-groups, mmol/L	0.352 (0.318-0.483)	0.320 (0.236-0.467)	0.258 (0.212-0.326)^a£^	0.001

Data are presented as arithmetic mean ± standard deviation, compared by ANOVA or as median (interquartile range), and compared by Kruskal-Wallis test. (a) Statistically significant compared to mild disease group. (b) Statistically significant compared to moderate disease group. (¥) *p* ≤ 0.05 was considered statistically significant (post hoc Tukey HSD). (£) *p* ≤ 0.017 was considered statistically significant (Mann–Whitney *U* test with Bonferroni correction).

**Table 3:** Significantly different parameters in the first point between deceased and recovered patients.

Parameter	Deceased patients *n* (18)	Recovered patients *n* (80)	*p*
Age, years	66 (47-74)	50 (40-61)	0.003
CRP, mg/L	71.30 (40.65-185.12)	9.75 (2.35-52.32)	<0.001
IL-6, pg/mL	22.70 (12.12-126.50)	5.15 (3.12-10.22)	<0.001
oxLDL, ng/mL	466 (366-714)	222 (196-293)	<0.001
TAS, mmol/L	753 (334-931)	1025 (872-1137)	0.002
TOS, mmol/L	12.5 (9.8-15.6)	24.6 (20.7-34.5)	<0.001
SOD, IU/L	101 (94-136)	140 (111-148)	0.001
PAB, U/L	67.8 (58.9-87.1)	75.73 (66.0-88.7)	0.004
MDA, *μ*mol/L	1.24 (1.17-1.55)	0.51 (0.40-1.20)	<0.001
SpO_2_, %	78 (66-86)	96 (94-97)	<0.001
LOS in hospital, days	21 (17-23)	10 (6-15)	<0.001
Oxygen supplementation, L/min	42 (30-55)	3 (0-15)	0.003

Data are presented as median (interquartile range) and compared by the Mann–Whitney *U* test.

**Table 4:** Association of oxLDL changes between the two-time points with the changes of other laboratory parameters.

	*Δ* oxLDL	
	Univariate model *β* (95% CI)	Multivariate model *β* (95% CI)
*Δ*TAS, mmol/L	0.150 (0.055-0.246) *p* = 0.002	0.130 (0.032-0.228) *p* = 0.010
*Δ*PAB, U/L	1.260 (0.295-2.226) *p* = 0.011	0.936 (-0.012-1.883) *p* = 0.053
*Δ*HDL 3c, %	-17.700 (-33.300-2.305) *p* = 0.025	-5.956 (-20.644-8.731) *p* = 0.421

CI: confidence interval; *Δ*: the changes between the first and second points.

## Data Availability

The data in this study could be made available upon reasonable request to the corresponding authors.
